# The Relationship Between Periodontal Disease and Nutrient Intake in Korean Adults: The Korea National Health and Nutrition Examination Survey (KNHANES VII) from 2016–2018

**DOI:** 10.3290/j.ohpd.b3240807

**Published:** 2022-07-22

**Authors:** Su-Yeon Hwang, Jung-Eun Park

**Affiliations:** a Researcher, Korean Dental Association Health Policy Institute, Seoul, Korea. Conceptualization, data curation, formal analysis, wrote, reviewed and edited the manuscript, had full access to all of the data in the study and takes responsibility for the integrity of the data and the accuracy of the data analysis, read and agreed to the published version of the manuscript.; b Assistant Professor, Department of Dental Hygiene, College of Health Science, Dankook University Cheonan, Chungcheongnam-do, Korea. Data curation, formal analysis, wrote, reviewed and edited the manuscript, had full access to all of the data in the study and takes responsibility for the integrity of the data and the accuracy of the data analysis, read and agreed to the published version of the manuscript..

**Keywords:** Korea National Health and Nutrition Examination Survey (KNHANES), nutrients, oral health, periodontal disease

## Abstract

**Purpose:**

To examine the association between the intake of various nutrients (phosphorus, riboflavin, thiamine, niacin, vitamin C, calcium, protein, carbohydrates, and fat) and the prevalence of periodontal disease in Korean adults.

**Materials and Methods:**

The data used for analysis were obtained from the 7th Korea National Health and Nutrition Examination Survey (2016–2018). Data from 12,689 adults aged ≥ 19 years who had a periodontal examination were analysed. Data were analysed using the Chi^2^ and t-tests. Multiple regression analysis was used to assess the association between the selected nutrients and periodontal diseases.

**Results:**

After adjusting for sex, age, income, body mass index, diabetes, smoking, alcohol consumption, and toothbrushing frequency, a statistically significant relationship between phosphorus, carbohydrate, and fat intake and the risk of periodontal disease was identified by multiple logistic regression analysis (odds ratio [OR]: 0.80, 95% confidence interval [CI]: 0.66–0.97; OR: 0.85, 95% CI: 0.70–0.98, OR: 1.41, 95% CI: 1.13–1.75, respectively).

**Conclusion:**

Phosphorus, carbohydrates and fat were associated with periodontal disease. Therefore, the improvement of diet should be emphasised to prevent and manage periodontal disease. Further research is needed based on various nutrients related to periodontal disease in the future.

Periodontal disease is a chronic inflammatory disease that occurs in the tissues around the teeth, and its associated symptoms such as alveolar bone loss, periodontal abscess, and tooth mobility can lead to tooth loss.^3,40^ In addition, the loss of masticatory function and a decrease in food intake negatively affect nutritional status, decreasing the patient’s quality of life.^16^

According to the 2018 Korea National Health and Nutrition Examination Survey (KNHANES) VII-3, the prevalence of periodontal disease nationwide was 23.4%, which was 26.4% lower than that reported in the 2015 survey.^26^ However, a quarter of the population still suffers from periodontal disease, which results in socioeconomic losses. Therefore, the systematic management of this condition is necessary.

Periodontal disease is caused by bacteria such as *Porphyromonas gingivalis* as well as host defense and environmental factors.^25,47^ The management of dental biofilm has mainly focused on reducing microbes in the oral cavity, which is necessary to eliminate periodontal disease and improve oral health. However, a multi-faceted approach is necessary because periodontal disease can be caused by other factors, such as smoking, diabetes, age, and nutrition.^29,36,38^ In particular, macronutrients derived from dietary intake provide crucial energy sources to maintain life. Micronutrients are necessary for the functionality of enzymes, structural moieties, and regulation of metabolic pathways.^14^

Adequate intake of nutrients can strengthen the body’s immune system, increasing resistance to infection, and positively influencing overall health, including the oral tissues. In contrast, insufficient nutritional intake is a significant cause of decreased immunity, affecting cell-mediated immunity, cytokine production, phagocyte functions, antibody response to mucosal secretion and complementary systems.^32^ Therefore, nutrition can play a major role in infections and inflammation, and malnutrition can favour infectious diseases by affecting innate and acquired immunity.^15^ In terms of the oral cavity, malnutrition has been reported to cause complications in acute necrotising ulcerative gingivitis (ANUG) and noma (cancrum oris).^17^

In addition, several studies have been performed on deficiency and excess of different nutrients and dietary control. Vitamin deficiencies or high carbohydrate intake have negatively impacted periodontal health.^42^ Sucrose, a carbohydrate, belongs to the disaccharides, which increases the growth of microorganisms in the oral cavity. Refined carbohydrates – such as sugar and white flour – promote nutritional imbalances in the oral microbiota and cause gingival and periodontal inflammation.^1,50^

In addition, some studies have reported a relationship between periodontal disease and diet. One study focused on reducing the incidence and severity of periodontal disease through a healthy diet and exercise regimen,^41^ another examined reducing inflammatory mediators in gingival tissue through calorie restriction and intermittent fasting.^39^ Also, diet type and dietary content can directly impact the oral microbiome. Previous studies have reported that alterations in dietary macronutrients can lead to a shift in the oral microbiome. In particular, excessive sugar intake can affect the metabolism of oral microbiota and promote the growth of acid-producing bacteria.^8,19^ Moreover, the progression of infection in the body causes an imbalance between catabolism and anabolism of the substances generated in response to the infection, adversely affecting nutritional status.^10^ As such, nutritional status and periodontal disease are closely related.

The literature contains several studies which investigated various approaches related to nutrients. However, only a limited number of studies exist on the association between periodontal disease and various nutrients in Korean adults. Therefore, this study examined the relationship between the intake of various nutrients (phosphorus, riboflavin, thiamine, niacin, vitamin C, calcium, protein, carbohydrates, and fat) and periodontal disease based on a sample survey of the Korean population.

## Materials and Methods

The survey protocols and secondary use of data were approved by the Institutional Review Board of the KDCA (IRB No. 2018-01-03-P-A).

### Study Participants

This study was performed using raw data from the KNHANES (VII) 2016–2018, conducted by the Korea Disease Control and Prevention Agency (KDCA). The study was approved by the KDCA Research Ethics Review Committee, and written informed consent was obtained from all participants (IRB No. 2018-01-03-P-A).

The KNHANES is a nationwide population-based survey of South Korea, and the data are open to the public.^26^ The primary sample units of the survey are selected through proportional allocation by a stratified, multistage, and probability sampling design. To ensure the extraction of representative samples of the population, the most recent Population and Housing Census data available at the time of the study were used as a basic framework for sampling. Study participants were selected from the data from dental examinations of KNHANES. The data of 13,199 were extracted from the 16,489 study participants, of which 12,689 who underwent periodontal tissue examination were included in this study.

### Periodontal Disease

The presence or absence of periodontal disease, a dependent variable, was determined using the Community Periodontal Index (CPI), according to the WHO-recommended standards.The CPI criteria were: 0: healthy and no signs of inflammation; 1: gingival bleeding; 2: calculus; 3: shallow pocket >3.5 mm and ≤5.5 mm; and 4: deep pocket; >5.5 mm. In this study, we defined a pocket depth <4 mm as normal (no) (CPI 0–2) and ≥4 mm as indicating periodontal disease (yes) (CPI 3, 4).^53^

### Nutrient Intake

Nutritional status was calculated based on each participant’s daily intake of nine nutrients: phosphorus, riboflavin, thiamine, niacin, ascorbic acid, calcium, protein, carbohydrate, and fat, which were assessed using a 24-h recall method. This consisted of collecting information about the type, amount, time of day, and location of food consumption by the respondent.

One day before the study, the food consumed by the participants, including ingredients and recipes, was recorded. Furthermore, a food model, a single-serving model for each food, and household weights and measures were used to aid the participant’s memory and accurately recall quantities. A multiple-pass approach was applied to minimise stress for the participants and promote accurate recall of food consumed.^21^ The individual nutrient intake was calculated using the raw data collect using this method.

### Covariates

The covariates of this study were the following sociodemographic factors: gender, age, income, BMI (Body Mass Index) , diabetes, smoking status, alcohol consumption, and tooth brushing/day.

Income was categorised into upper, middle, and lower incomes. BMI < 18.5 was classified as underweight, 18.5–22.9 as normal weight, 23–24.9 as overweight, and >25 as obese. Diabetes was classified as fasting blood sugar ≥126 mg/dl as diagnosed by a physician, or taking diabetes medication or insulin injection.^52^ Smoking was divided into the following categories: lifetime consumption <5 packs, ≥5 packs, and non-smokers. Alcohol consumption was defined as taking more than one drink per month in the past year; all other cases were defined as non-drinking.^23^ Finally, the frequency of toothbrushing per day was categorised into three groups: <1, <2 or ≥3 times per day.

### Statistical Analysis

The strata (kstrata) used in the data analysis of the KNHANES is an integrated design layer to estimate variance, and the cluster (psu) corresponds to the primary extraction unit when designing a sample. Moreover, weight was applied to analyse the data via complex sampling. A Chi^2^ test was performed on the correlation between the participant’s general characteristics and the prevalence of periodontal disease, and a t-test was used to evaluate the relationship betweeen nutrient intake and the occurrence of periodontal disease. Subsequently, after dividing the nutrient intake by quartile, univariate and multivariate logistic regression analyses were performed to test the relationship between nutrient intake and periodontal disease. In this case, confounding variables that may affect the relationship were corrected and analysed. All statistical analyses conducted in this study were performed using SPSS Statistics for Windows, Version 20.0 (IBM; Armonk, NY, USA), and the statistical significance test was based on a type-1 error level of 0.05.

## Results

The data from the KNHANES VII survey can be accessed and downloaded from the KNHANES homepage (URL:https://knhanes.kdca.go.kr/knhanes/sub03/sub03_02_05.do accessed on 19 May 2022).

### General Characteristics of Participants

The results of periodontal disease according to the general characteristics of the study subjects are shown in Table 1. Among adults aged ≥19 years, a higher prevalence periodontal disease was found in men (37.7%) than in women (25.3%). In adults ≥19 years of age, periodontal disease was identified in 38.4% of those aged in their 50s and 47.0% of those aged in their 60s, suggesting an increased prevalence with age. In terms of the participant’s income level, 34.3% of subjects in with low income and 30.3% of those of the mid-income level had periodontal disease, suggesting a greater prevalence with lower income. It was shown that the prevalence of periodontal disease was greater with increased weight, especially with obesity, and 52.0% of subjects diagnosed with diabetes had a higher periodontal disease rate. Periodontal disease was observed in 40.7% of the subjects who smoked ≥5 packs of cigarettes during their lifetime, and 31.2% of non-drinkers had periodontal disease. A higher prevalence of periodontal disease was observed in subjects who brushed their teeth ≤ once per day (43.9%).

**Table 1:** General characteristics of subjects by periodontal diseases

Characteristics		Periodontal disease		Total n (%)	p**
		No n (%)*	Yes n (%)*		
Sex	Man	3417 (62.3)	2128 (37.7)	5545 (100.0)	<0.001
	Woman	5291 (74.7)	1853 (25.3)	7144 (100.0)	
Age	19–29	1501 (96.0)	61 (4.0)	1562 (100.0)	<0.001
	30–39	1806 (86.5)	294 (13.5)	2100 (100.0)	
	40–49	1768 (74.8)	647 (25.2)	2415 (100.0)	
	50–59	1466 (60.6)	1005 (39.4)	2471 (100.0)	
	≥60	2167 (53.0)	1974 (47.0)	4141 (100.0)	
Income	Lower	2052 (65.7)	1092 (34.3)	3144 (100.0)	<0.001
	Median	4386 (69.7)	2002 (30.3)	6388 (100.0)	
	Upper	2252 (72.8)	872 (27.2)	3124 (100.0)	
BMI	Underweight	379 (81.9)	96 (18.1)	475 (100.0)	<0.001
	Normal	3523 (75.0)	1210 (25.0)	4733 (100.0)	
	Overweight	1839 (65.9)	955 (34.1)	2794 (100.0)	
	Obesity	2760 (64.1)	1628 (35.9)	4388 (100.0)	
Diabetes	Absence	8134 (71.6)	3358 (28.4)	11492 (100.0)	<0.001
	Presence	574 (48.0)	623 (52.0)	1197 (100.0)	
Smoking status	<5packs	216 (84.9)	40 (15.1)	256 (100.0)	<0.001
	≥5packs	2721 (59.3)	1918 (40.7)	4639 (100.0)	
	Non–smoker	5699 (74.7)	1978 (25.3)	7677 (100.0)	
Alcohol drinking	Non–drinker	3921 (68.8)	1830 (31.2)	5751 (100.0)	0.22
	Drinker	4716 (70.0)	2113 (30.0)	6829 (100.0)	
Tooth brushing/day	≤1	611 (56.1)	470 (43.9)	1081 (100.0)	<0.001
	2	3196 (66.4)	1660 (33.6)	4856 (100.0)	
	≥3	4900 (73.6)	1850 (26.4)	6750 (100.0)	

* Weighted %; ** p–value was calculated by complex sample Chi^2^ test.

### Relationship Between Nutrient Intake and Periodontal Disease

The nutrient intake level according to the presence or absence of periodontal disease is shown in Table 2. Of the nine nutrients examined, the periodontal disease group was found to consume less phosphorus (1029.45 ± 514.10 mg/day), riboflavin (1.44 ± 0.91 mg/day), niacin (12.62 ± 8.20 mg/day), vitamin C (59.81 ± 65.84 mg/day), calcium (503.10 ± 333.52 mg/day), protein (66.32 ± 39.03 g/day), and fat (36.76 ± 26.00 g/day) than the normal group. Among other nutrients, there was no statistically significant between-group difference in thiamine intake, and a mean of 9.46 g more carbohydrates (301.25 ± 130.44 g/day) was consumed in the periodontal group than in the normal group.

**Table 2:** Intake of various nutrients according to occurance of periodontitis (mean ± SE)

Nutrients	Periodontal disease		Range	p*
	No	Yes		
Phosphorus intake (mg)/day	1053.13 ± 496.81	1029.45 ± 514.10	4–6461	<0.001
Riboflavin intake (mg)/day	1.56 ± 0.89	1.44 ± 0.91	0–11	<0.001
Thiamine intake (mg)/day	1.30 ± 0.75	1.30 ± 0.74	0–12	0.020
Niacin intake (mg)/day	13.27 ± 7.86	12.62 ± 8.20	0–215	0.533
Vitamin C intake (mg)/day	62.65 ± 70.32	59.81 ± 65.84	0–1273	0.860
Calcium intake (mg)/day	510.98 ± 310.35	503.10 ± 333.52	1–5555	0.375
Protein intake (g)/day	70.39 ± 39.06	66.32 ± 39.03	0–682	<0.001
Carbohydrate intake (g)/day	291.79 ± 122.66	301.25 ± 130.44	6–1837	<0.001
Fat (g)/day	44.82 ± 34.44	36.76 ± 26.00	0–514	<0.001

*t-test.

### Logistic Regression Analysis of Nutritional Factors Affecting Periodontal Disease

The odds ratios for periodontal disease in each intake group based on the upper intake group are listed in Table 3. The odds ratio of the periodontal diseases gradually increased as they moved down to the lower intake group of riboflavin (Quartile 1 and 2), which was statistically significant (p < 0.05). The periodontal disease odds ratio for niacin intake was increased 1.37-fold in Quartile 1, and the periodontal disease odds ratio for protein intake was increased 1.34-fold in Quartile 1 (p < 0.05). The periodontal disease odds ratio for fat intake showed a 2.42-fold, statistically significant increase in Quartile 1 compared to Quartile 4 (p < 0.05).

**Table 3:** Relationship between intake of various nutrients and periodontal disease: logistic regression analysis

Quartile of nutrition intake^a^	Model 1				Model 2			
	OR (95% CI)				OR (95% CI)			
	Q1	Q2	Q3	Q4	Q1	Q2	Q3	Q4
Phosphorus	1.11 (0.96–1.28)	0.96 (0.84–1.10)	0.92 (0.81–1.06)	Reference	0.81 (0.55–1.17)	0.77 (0.58–1.03)	0.80 (0.66–0.97)^*^	Reference
Riboflavin	1.51 (1.31–1.74)^*^	1.21 (1.06–1.38)^*^	1.05 (0.92–1.19)	Reference	1.05 (0.80–1.37)	1.09 (0.88–1.35)	1.00 (0.85–1.17)	Reference
Thiamine	0.93 (0.82–1.07)	0.95 (0.83–1.08)	1.03 (0.91–1.17)	Reference	0.85 (0.66–1.07)	0.88 (0.73–1.06)	0.99 (0.84–1.16)	Reference
Niacin	1.37 (1.19–1.57)^*^	1.10 (0.96–1.26)	1.11 (0.97–1.27)	Reference	1.20 (0.94–1.56)	1.04 (0.84–1.27)	1.14 (0.96–1.36)	Reference
Vitamin C	1.08 (0.98–1.24)	1.08 (0.94–1.24)	1.07 (0.94–1.22)	Reference	1.09 (0.91–1.31)	1.07 (0.90–1.26)	1.04 (0.90–1.20)	Reference
Calcium	1.09 (0.95–1.26)	1.07 (0.94–1.22)	0.98 (0.86–1.11)	Reference	0.98 (0.77–1.24)	1.06 (0.88–1.27)	0.98 (0.84–1.15)	Reference
Protein	1.34 (1.17–1.54)^*^	1.14 (0.99–1.31)	1.07 (0.96–1.22)	Reference	1.32 (0.94–1.86)	1.14 (0.89–1.54)	1.11 (0.91–1.35)	Reference
Carbohydrate	0.81 (0.70–0.93)	0.85 (0.75–0.97)	0.94 (0.83–1.06)	Reference	0.85 (0.68–1.00)	0.85 (0.70–0.98)^*^	0.95 (0.82–1.01)	Reference
Fat	2.42 (2.11–2.77) ^*^	1.70 (1.50–1.92)^*^	1.40 (1.22–1.60) ^*^	Reference	1.41 (1.13–1.75) ^*^	1.26 (1.04–1.53)^*^	1.19 (1.00–1.41) ^*^	Reference

Data are presented as OR (95% CI). OR: odds ratio; CI: confidence interval, *p<0.05. Model 1: unadjusted model; model 2: adjusted for factors (sex, age, and household income, BMI, diabetes, smoking, alcohol consumption, and toothbrushing). A quartile of nutrition intake was classified as <25% (the lowest quartile group, Q1), 25–49% (Q2), 50–74%(Q3), and 75–100% (the highest quartile group, Q4).

After adjusting for risk factors of periodontal diseases such as age, income level, BMI, diabetes, smoking, alcohol consumption, and toothbrushing frequency, phosphorus intake was associated with periodontal disease in Quartile 3 (OR: 0.80; 95% CI 0.66–0.97, p < 0.05). Finally, carbohydrate intake decreased by 0.85-fold (95% CI 0.70–0.98) in Quartile 2 (p < 0.05). Furthermore, the odds ratio of periodontal disease for fat intake showed a significant 1.41-fold (95%CI 1.13–1.75) increase in Quartile 1.

## Discussion

Periodontal disease is caused by the reaction between dental biofilm in the oral cavity and the host, with various factors contributing to it.^48^ Periodontal disease may be affected by genetic factors, the environment and microorganisms, but may also result from various other risk factors. Nutrient intake is essential for all organisms, with large variations between individuals. In this study, the analysis was conducted based on data from the KNHANES VII to understand the intake of various nutrients and the associated risk factors according to the presence or absence of periodontal disease.

The morbidity rate of periodontal disease was significantly higher in men, older people, those with diabetes, and smokers. These results agree with those of previous studies.^5,12,18,29,51^ The prevalence of periodontal disease also appeared to increase proportionally to increased BMI. Therefore, the morbidity of periodontal disease was higher in the overweight and obese groups than in the normal weight group, similar to the findings of a previous study.^51^ In addition, lower income,^46^ frequency of toothbrushing and alcohol abstinence also influenced the prevalence of periodontal disease to a similar degree.^24,28^ Therefore, the association between the participants’ demographics and the presence of periodontal disease found in this study is consistent with the results of previous studies.

In most cases, the intake levels of the nutrients analysed in this study were high in the periodontally healthy group. There was no difference in thiamine intake between the periodontal disease group and the periodontally healthy group (p < 0.05), and carbohydrate intake was higher in the periodontal disease group than in the healthy group (p < 0.001).

Micronutrients are known to benefit periodontal tissues by acting as antioxidants and affecting bones and muscles; thiamine (B1) converts sugar into energy for normal muscle and nerve function,^35^ and riboflavin (B2) develops muscles related to growth.^27^ The vitamin B complex has also been reported to benefit human tissue wound healing.^37^ In addition, vitamin C deficiency may weaken the immune response^49^ and cause abnormalities in collagen synthesis, which may eventually affect the growth and repair of periodontal tissues.^2^

Phosphorus affects the remineralisation of bones and teeth by interacting with calcium, magnesium, and vitamin D,^54^ and calcium is a nutrient that assists hard tissues. It has been reported that periodontal disease indices such as probing depth, bleeding, and the gingival index appear better in participants supplemented with dietary calcium, suggesting that periodontal tissues may be affected by calcium.^33^ It has also been reported that protein increases the fractional absorption of dietary calcium and contributes to bone mineralisation.^9^ In addition, protein is known to influence the impairment of immune parameter response to pathogens and reduce the impact on the immune system.^32^ Considering these effects, protein is thought to protect the periodontal tissues.

In the upper intake group for each nutrient, as the intake of riboflavin, niacin, and protein decreased, the risk of periodontal disease increased by 1.51, 1.37 and 1.34 times, respectively, which was statistically significant. However, the nutrient effects were not statistically significant (p > 0.05) in an adjusted model after correcting for several confounding factors.

The risks of periodontal disease statistically significantly decreased 0.8-fold in the upper-median phosphorus intake group (Quartile 3) and 0.85-fold in the lower-median carbohydrate-intake group (Quartile 2), but increased 1.41-fold in the lower fat-intake group (Quartile 1) after correcting for confounding factors.

Phosphorus is a nutrient that potentially interacts with calcium, magnesium, and vitamin D, which affects the bones and hard tissues. It has been reported that the concentration of phosphorus and calcium in the dental biofilm or saliva can affect the balance between enamel demineralisation and remineralisation.^54^ Therefore, these nutrients are expected to positively affect the mineralisation of alveolar bone and other oral hard tissues as well as the tooth surface. In a study examining the relationship between the daily intake of calcium, phosphorus, magnesium, and the calcium:phosphorus ratio and dental caries in the child population, only the calcium:phosphorus ratio was statistically significantly inversely proportional to the caries index.^30^ Based on these results, phosphorus can be considered a factor that affects the interactions of various nutrients that affect bones and hard tissues. The proper balance of calcium and phosphorus is considered essential to maintain optimal bone density and dental health.

Studies in which sugars and carbohydrates are involved in dental caries have been conducted for many years. Furthermore, it has been reported that sugar can increase inflammation and oxidative stress that cause inflammatory conditions in periodontal disease, type 2 diabetes, and cardiovascular disease.^44^ In addition, it can also act as a systemic risk factor for visceral fat accumulation, dyslipidemia, and insulin resistance,^13,20,34^ which can eventually contribute to the inflammatory process of periodontal tissue. It has also been reported that obesity, an indicator of excessive consumption of fermentable carbohydrates, is associated with an increased risk of periodontal disease.^43^

High sugar intake may act as a local factor of oxidative stress and the growth of intraoral biofilms, resulting in bacterial imbalance.^11^ Biofilms also increased four weeks after diet control and oral hygiene management without refined sugars, but gingival bleeding and probing depth decreased.^6^ A high carbohydrate or sugar intake level could be a potential factor in gingival and periodontal diseases, as studies have shown that local gingivitis is mild in the absence of refined sugar intake. This study also showed a similar result because the presence or absence of periodontal disease was classified based on the periodontal pocket measurement test using the CPI.

A study analysing the relationship between sugar intake and periodontal disease in the youth population using raw data from NHANES III (1988–1994) in the USA found that high sugar consumption is related to periodontal disease and traditional risk factors.^31^ That study also reported that high sugar consumption can contribute to the systemic inflammation observed in non-infectious diseases related to periodontal disease.^31^ This study also confirmed that the risk of periodontal disease was reduced 0.85-fold in the lower carbohydrate intake Quartile 2 group (p < 0.05).

Finally, in this study, fat intake was associated with the prevalence of periodontal disease. Previous studies reported that an abundant intake of omega-3 fatty acids had an anti-inflammatory effect on periodontal tissues.^7,22^ It has been reported that in *P. gingivalis*-induced periodontal disease, the intake of omega-3 fatty acids regulated inflammatory cytokine/mediator messenger RNA expression, thus showing a positive effect on periodontal tissues. Therefore, it was suggested that a diet high in omega-3 fatty acids might serve as an effective prevention and intervention strategy for periodontal disease.^7,22^

However, most studies have reported that a high-fat diet showed a negative effect on periodontal disease due to oxidative stress.^4,47^ In the KNHANES data used in this study, the classified nutrients items do not include omega-3 fatty acids and do not differentiate types of fat. Therefore, it is clear that a more in-depth investigation of the association between fat and periodontal disease is needed. Research on different types of fat should be conducted to improve understanding of the association between fat and periodontal disease.

The limitations of the present study are outlined as follows: First, there was no subcategorisation of macronutrients in the KNHANES sampling data used in this study. Therefore, results according to further classification of carbohydrates (simple, complex, fibrous), protein (plant, animal), and fat (saturated, unsaturated) could not be presented, which limits the accuracy of statements about these macronutrients. Second, certain micronutrients, such as vitamin D and magnesium, were not included in the KNHANES sampling data. Therefore, there was a limit to understanding the relationship and interaction between micronutrients and periodontal disease. Third, in the KNHANES, consumption of antibiotics was not an exclusion criterion, and oral prophylaxis practices that may affect periodontal disease were not considered. Therefore, in this study, the relationship of toothbrushing frequency per day was considered. Fourth, as this study was cross-sectional, causal relationships could not be identified. Nevertheless, the relationship between various nutrients and periodontal disease was investigated using KNHANES data, which are considered representative samples of Korean adults. Longitudinal studies on various nutrients and periodontal disease are needed. Also, further studies on the interaction of micronutrients such as phosphorus, calcium, magnesium, vitamin D and omega-3 fatty acids need to be conducted.

## Conclusion

The intake of phosphorus, carbohydrates, and fat was associated with periodontal disease. Therefore, improving overall dietary habits should be emphasised to prevent and manage periodontal disease. Further research is needed based on various nutrients related to periodontal disease.
